# Subcutaneous nodules and dermatitis associated with non-*immitis* non*-repens* dirofilariosis morphologically consistent with *Dirofilaria striata* in a 2-year-old male domestic cat in Florida, USA

**DOI:** 10.1080/01652176.2020.1814972

**Published:** 2020-09-08

**Authors:** Danielle Wyatt, Domenico Santoro, Kelly Deabold, Jeff Gruntmeir, April Childress, William F. Craft, Heather D. S. Walden, James F. X. Wellehan

**Affiliations:** a Department of Small Animal Clinical Sciences, College of Veterinary Medicine, University of Florida, Gainesville, FL, USA; b Department of Comparative, Diagnostic and Population Medicine, College of Veterinary Medicine, University of Florida, Gainesville, FL, USA

**Keywords:** Cat, feline, *Dirofilaria*, *Dirofilaria striata*, cutaneous nodule, macrocyclic lactone, moxidectin

A 2-year-old, neutered male domestic shorthair cat was adopted as a stray in Florida, USA. At adoption, the cat was treated for fleas (spinosad; Comfortis^®^, Elanco, Greenfield, IN, USA) by the primary veterinarian. One month after adoption, the cat developed two pea-sized (approximately 10 × 10 mm) subcutaneous non-alopecic nodules along the right lumbar spine and left clavicle. After a physical examination, the veterinarian collected blood for a complete blood count and serum biochemistry panel. Bloodwork revealed moderate eosinophilia (1,536 G/L; range: 0–1,000 G/L) and mild elevation of blood urea nitrogen (2.3 mmol/L; range: 0.83–2.14 mmol/L). All other parameters were within the normal reference ranges. Serology did not detect *Dirofilaria immitis* antigen, feline immunodeficiency virus antibody, or feline leukemia virus p27 antigen (Snap^®^ Feline Triple^®^ Test, IDEXX, Westbrook, ME, USA). Other health concerns were not noted at that time. A fine needle aspirate (FNA) was performed by the veterinarian showing a heterogeneous lymphoid population. Skin biopsies were also taken from the two nodules and it showed granulomatous and eosinophilic dermatitis with presumptive intralesional nematodes. Over the following month, additional sub-cutaneous nodules appeared, and the cat was referred to the Dermatology Service – Small Animal Hospital at the University of Florida – College of Veterinary Medicine for evaluation.

**Figure 1.**
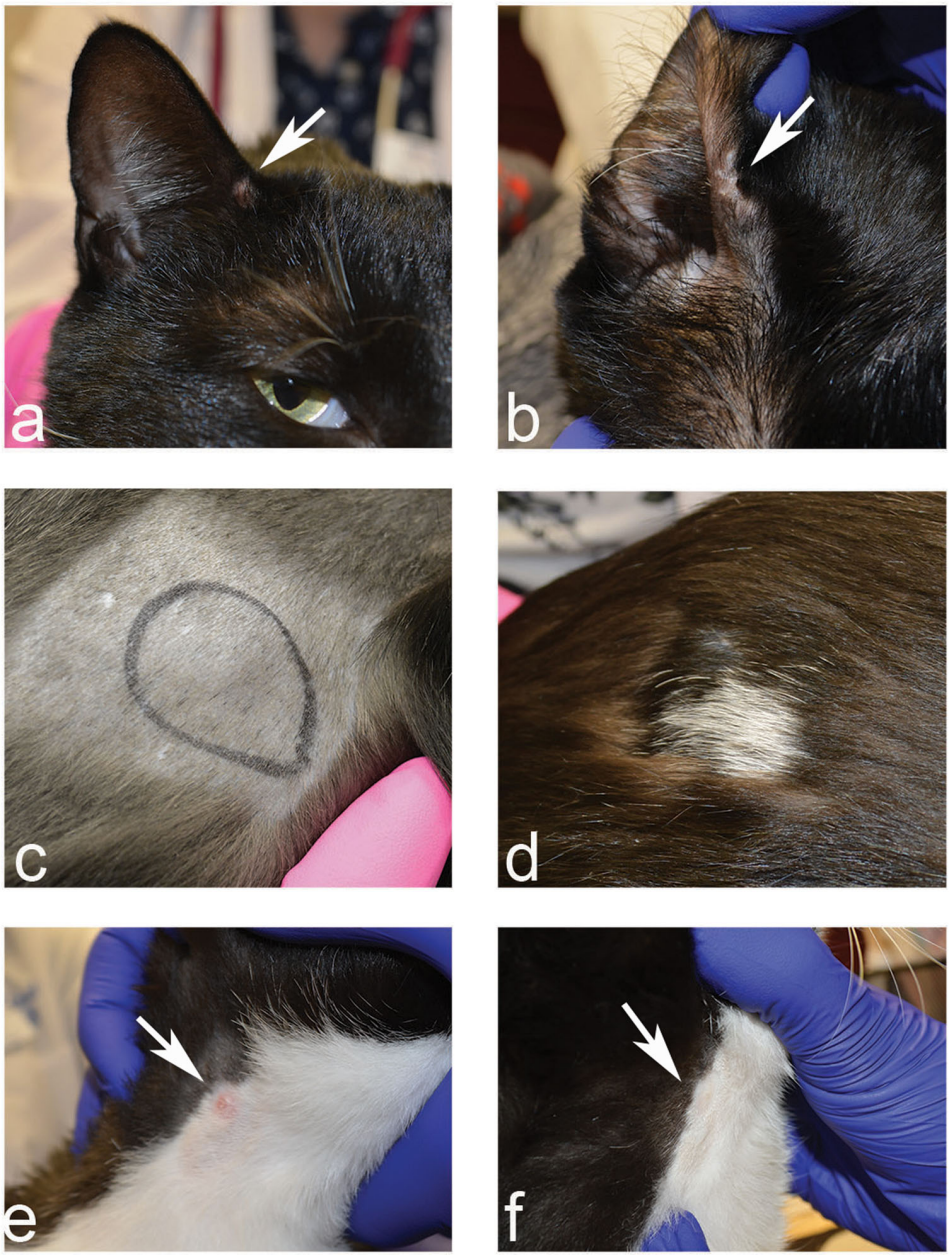
Clinical photographs before and after treatment of a 2-year-old, neutered male domestic shorthair cat with subcutaneous nodules and dermatitis. Note the dermal nodules on the cat’s pinna (a), flank (c), and lateral neck (e) at presentation. Two months after surgical removal and topical application of 10% imidacloprid/1% moxidectin, a significant improvement with lack of reoccurrence was noted at the same location (pinna [b], flank [d], and lateral neck [f]). The circle in figure (c) highlights the dermal nodule.

Upon arrival the cat was bright and alert. He weighed 5.58 kg with a body condition score of 4/9 (Bjornvad et al. 2011) and a temperature of 38.9 °C (reference range of 36.7–38.9 °C) (Levy et al. 2015). The cat presented with multiple cutaneous nodules on the concave aspect of the right pinna (0.5 × 0.4 cm), the dorsal and ventral neck (0.4 × 0.4 cm and 0.5 × 0.4 cm, respectively), and the right hip (1 × 0.7 cm) (Figure 1). The pre-scapular and popliteal lymph nodes were symmetrical and mildly enlarged and showed a mixed population of lymphocytes that were indicative of reactive inflammation. An air-dried Wright Giemsa stained blood smear was also examined and showed no evidence of parasitic or blood-borne microorganisms.

**Figure 2.**
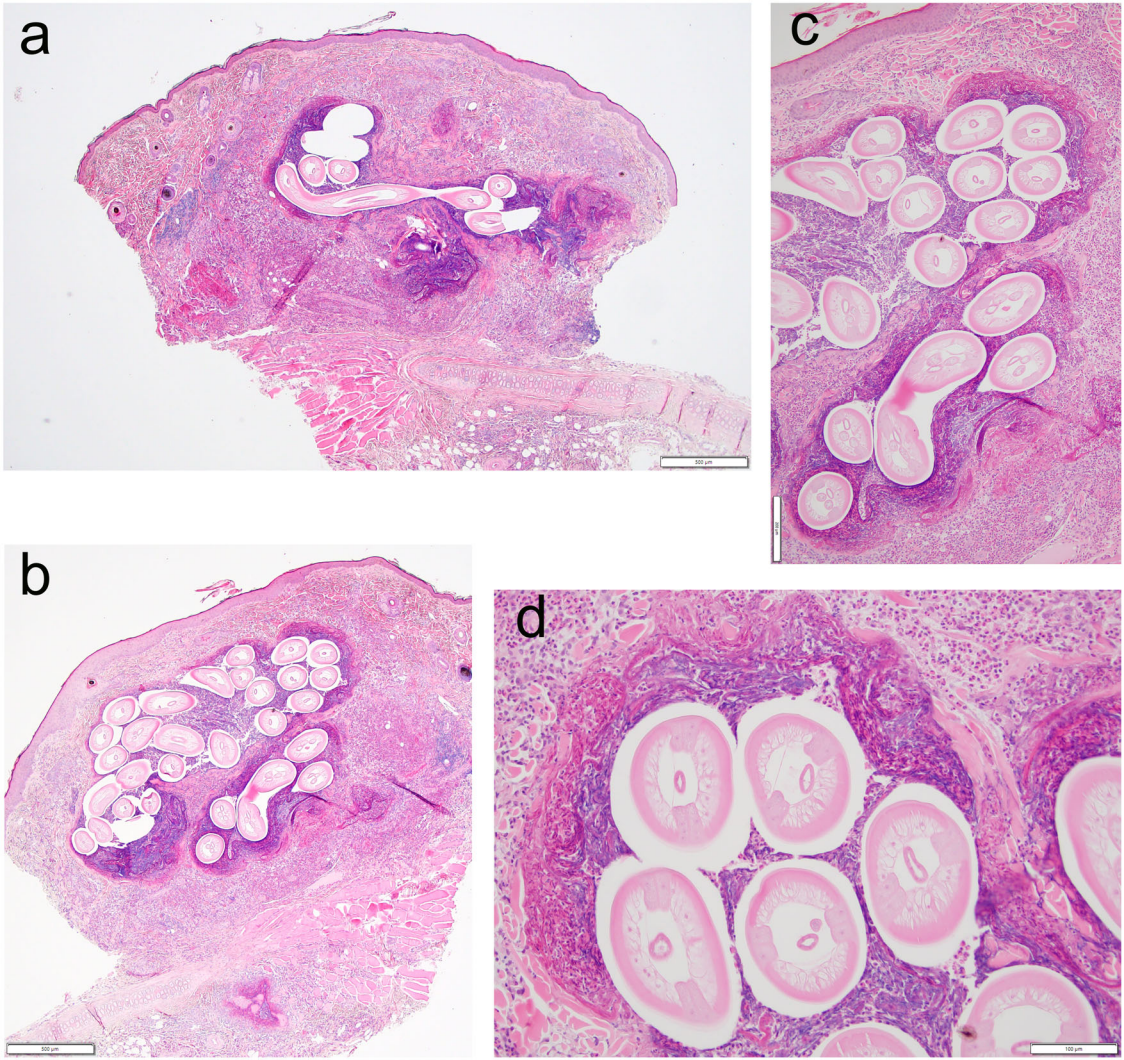
Haired skin from the pinna of a 2-year-old male domestic shorthair cat. Nodular to diffuse eosinophilic, mastocytic, and histiocytic dermatitis and panniculitis associated with many cross and longitudinal sections of nematode larvae and adults within dermal migration tracts. 40x magnification (a & b) (Scale bar: 500 µm); 100x magnification (c) (Scale bar: 200 µm); 200x magnification (d) (Scale bar: 100 µm).

Excisional biopsies were obtained from the right hip and dorsal neck, and a shave biopsy was obtained from a pinnal nodule. The right hip biopsy was bisected, and one-half was placed in saline and submitted to the parasitology laboratory. The remaining samples were submitted for histopathologic examination. The histopathology revealed a nodular to diffuse, eosinophilic, histiocytic, and mastocytic dermatitis and panniculitis with collagen flame figures, and many nematode larvae and adults within nodules and migration tracts (Figure 2a–d). The nematodes were approximately 150–250 µm in diameter, and had an approximately 5–10 µm thick, eosinophilic, refractive smooth external cuticle. The nematodes also had polymyarian-coelomyarian musculature lining a pseudocoelom, prominent lateral cords with internal cuticular ridges, small intestinal tracts lined by single layers of cells, and occasionally contained female and male reproductive organs (larvae and adult nematodes) (Figure 2a–d). Concurrently, a focal sarcocyst was found on the dorsal neck.

**Figure 3.**
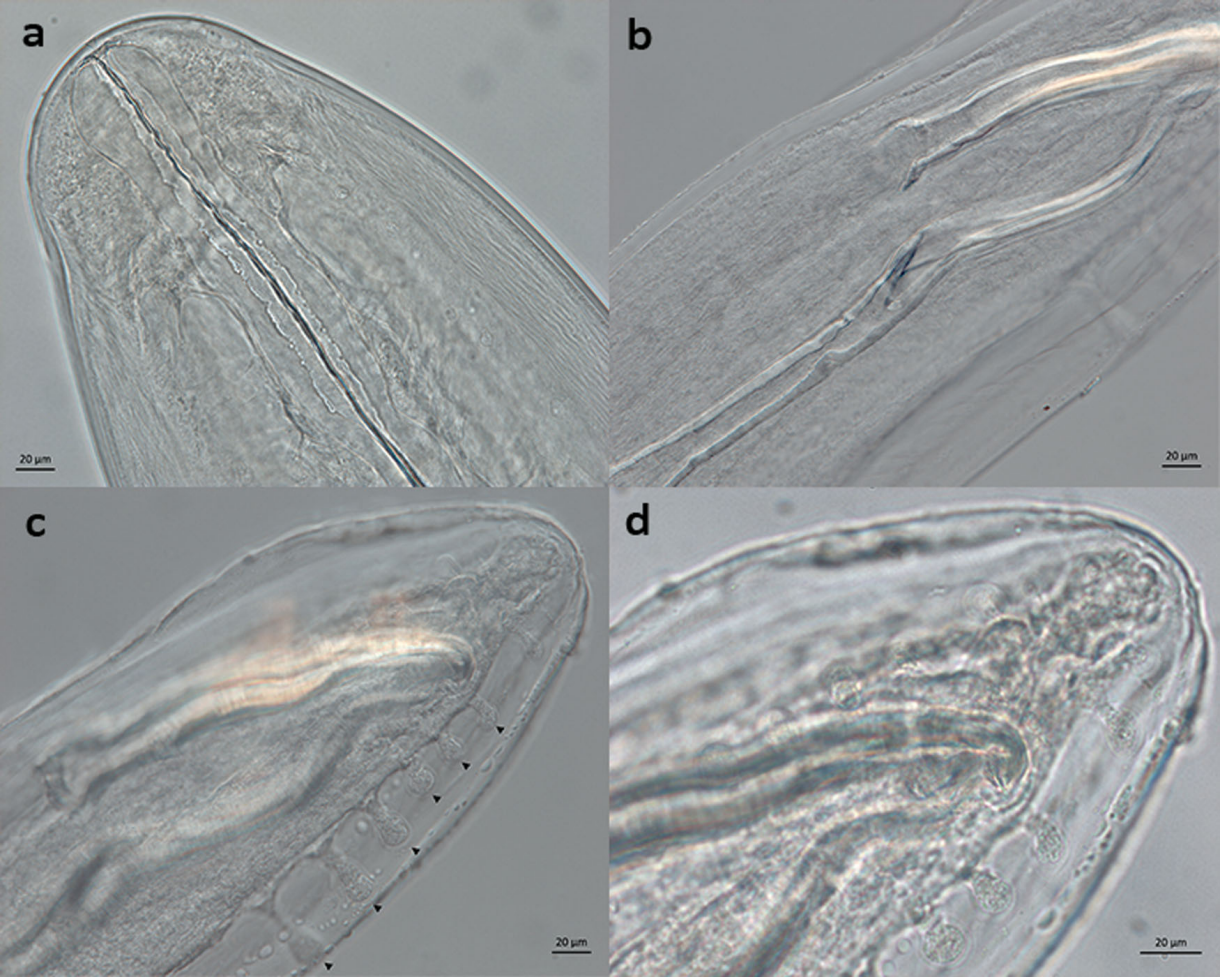
Suspected adult *Dirofilaria striata* male and female fragments recovered from feline skin biopsy sample. (a) Anterior end of adult female 400x magnification; (b) Unequal spicules of male posterior, junction of left spicule, 400x magnification; (c) Male posterior six pedunculate preanal papillae (arrowheads), 400x magnification; (d) Tip of right spicule, 630x magnification. Scale bar: 20 µm.

The parasitological examination of biopsy samples by maceration of tissue and saline sedimentation revealed fragments of adult male and female nematodes with the anterior end consistent with *Dirofilaria* spp. (Figure 3a–d). A single male posterior fragment was recovered and was consistent with *D. striata* (Figure 3b–d) (Sonin 1985). The male posterior end had six pedunculate preanal papillae and heavily sclerotized, unequal spicules measuring 250.23 µm (right) and 421.38 µm (left). Serum was used for heartworm antigen testing with and without heat treatment (Little et al. 2014) by the WITNESS™ Canine Heartworm Antigen Test (Zoetis, Florham Park, NJ, USA) and without heat treatment by the SNAP^®^ Feline Triple^®^ Test (IDEXX, Westbrook, ME, USA) and no *D. immitis* antigen was detected. A modified Knott’s microfilariae examination was performed on 2 mL of whole blood, and microfilariae were not recovered. Serology was positive for *D. immitis* antibody (Solo Step^®^ FH immunoassapy, HESKA; Loveland, CO, USA) and rare *Ancylostoma* spp. ova were seen following centrifugal fecal flotation (Sheather’s sugar; specific gravity 1.25). An echocardiogram and three-view thoracic radiographs were also performed and did not show abnormalities in the pulmonary artery. An ophthalmic examination for larvae or adults nematodes of *D. immitis* was also performed and was unremarkable.

DNA was extracted from a worm fragment using a commercial kit (DNeasy tissue kit, Qiagen, Valencia, CA, USA). Polymerase chain reaction (PCR) amplification of the nematode 18S rRNA gene was done as previously described (Floyd et al. 2005), but PCR was not performed on venous blood from the cat (to assess microfilarial nucleic acid). PCR amplification of nematode mitochondrial cytochrome oxidase subunit I (COI) and 12S rRNA genes was also done as previously described (Casiraghi et al. 2001). The PCR products were resolved in 1% agarose gels, and the bands of interest were excised and purified using a gel extraction kit (QIAquick gel extraction kit, Qiagen, Valencia, CA, USA). Direct sequencing was performed using a Big-Dye Terminator Kit (Perkin-Elmer, Branchburg, NJ, USA) and analyzed on ABI automated DNA sequencers. All products were sequenced in both directions. Primer sequences were edited out prior to further analyses. After primers were edited out, the 18S rRNA gene product was 822 base pairs (bp), the COI gene product was 626 bp, and the 12 s rRNA product was 412 bp. The sequences were submitted to GenBank under accession numbers MN635455 - MN635457.

The sequences were compared to those in GenBank (National Center for Biotechnology Information, Bethesda, Maryland, USA), EMBL (Cambridge, United Kingdom), and Data Bank of Japan (Mishima, Shizuoka, Japan) databases using BLASTN (Altschul et al. 1990). BLASTN searches found that the 18S rRNA gene product was 94.31% homologous with *D*. *repens* (GenBank accession # MK192092) and 96.84% homologous with *D. immitis* (AB973231), the COI gene product was 92.65% homologous with *D*. *repens* (GenBank # KR998259) and 90.73% homologous with *D. immitis* (AJ537512), and the 12S gene product was 88.38% homologous with *D*. *repens* (AJ544832) and 90.91% homologous with *D. immitis* (MH051846).

**Figure 4.**
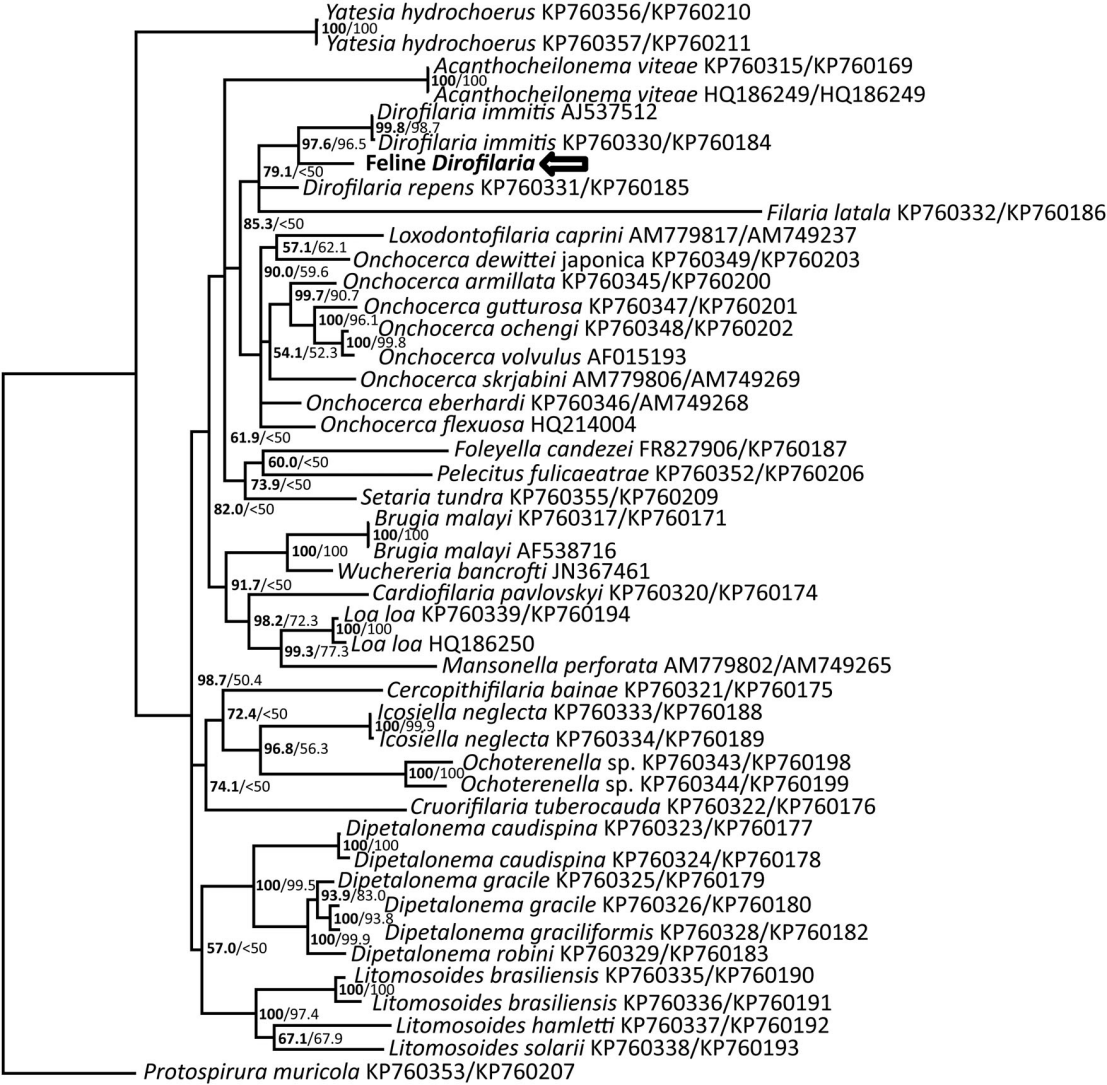
Bayesian phylogenetic tree of MAFFT alignment of concatenated homologous mitochondrial cytochrome oxidase I and 12S rRNA genes of representative Filarioidea. *Protospirura muricola* was used as the outgroup. Confidence of the tree topology obtained is shown by Bayesian posterior probabilities to the left of the slash or above, and maximum likelihood bootstrap values are to the right or below. The probable *Dirofilaria striata* is marked with an arrow.

Predicted homologous filarid nucleotide sequences of the 18S rRNA, COI, and 12S rRNA genes were aligned using MAFFT (Katoh and Toh 2008). Molecular sequence data for *D. striata* was previously unavailable. *Protospirura muricola*, in the Spriuridae (GenBank accession numbers KP760353 and KP760207), was used as an outgroup. Bayesian analyses of nucleotide alignments were performed using MrBayes 3.2.6 on the CIPRES server, with gamma distributed rate variation and a proportion of invariant sites, and a general time reversible substitution model (Ronquist et al. 2012; Miller et al. 2015). The first 25% of 2,000,000 iterations were discarded as a burn in. All genes showed similar phylogeny, but a number of taxa lacked representative 18S data, so COI and 12S sequences were concatenated for the Bayesian tree shown (Figure 4). The analysis found 97.6% posterior probability for this organism clustering with *D*. *immitis*, at a distance consistent with a separate species. Clustering of this organism and *D*. *immitis* with *D. repens* and *Filaria latala* had a 79.1% posterior probability.

Maximum likelihood analysis of the alignment was performed using RAxML on the CIPRES server, using a gamma distributed rate variation, a proportion of invariant sites, and a general time reversible model (Stamatakis et al. 2008). Again, *Protospirura muricola* was used as an outgroup. To test the strength of the tree topology, bootstrap analysis was used (1000 re-samplings) (Felsenstein 1985). ML bootstrap values are shown on the Bayesian tree (Figure 4). The analysis found a 96.5% bootstrap value for this organism clustering with *D*. *immitis*. Clustering of this organism and *D*. *immitis* with *D. repens* had a 72.7% posterior probability, although the ML analysis did not place *Filaria latala* with this clade.

**Figure 5.**
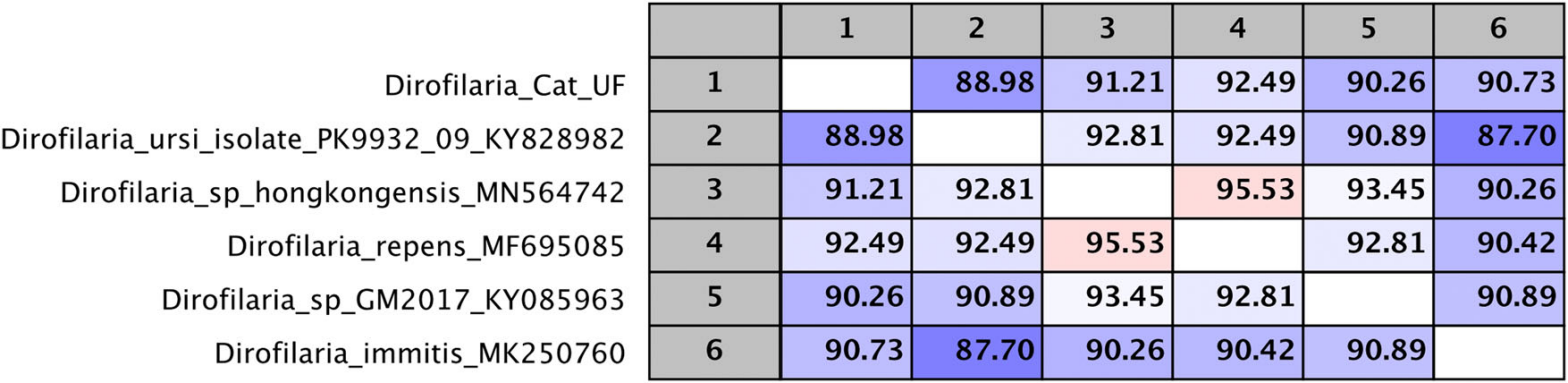
Table of percent nucleotide homology of a MAFFT alignment of COI sequences. The probable *Dirofilaria striata* is named Dirofilaria_cat_UF.

Additional reference sequences for the COI gene were available for *D. lutrae*, *D. ursi*, *Dirofilaria* sp. ‘hongkongensis’, and *Dirofilaria* sp. ‘GM2107’ from a dog in Argentina, but additional 18S and 12S rRNA sequences were not available. High confidence of branching order was not found in Bayesian and ML analyses based solely on COI sequence, but a table of percent nucleotide homology of a MAFFT alignment of COI sequences shows that our sequence is distinct from other *Dirofilaria sp*. at a distance consistent with a novel species (Figure 5).

The morphological analysis was consistent with *D. striata.* The molecular analysis revealed that this organism was distinct from other known *Dirofilaria* sp. We were unable to locate any reference specimens of *D. striata*. We have unsuccessfully attempted to locate a specimen with intact DNA from a wild felid in the southeastern United States. However, even if a specimen is obtained, without a reference specimen, there may not be a more definitive way to identify *D. striata* with currently available diagnostic methods.

Oral (PO) doxycycline (10 mg/kg BW q24 hours) was initiated based upon published guidelines for the treatment of feline heartworms (Jones et al. 2014). However, doxycycline had to be discontinued 7 days later due to anorexia. The doxycycline dose was split (5 mg/kg BW PO q12 hours) but the cat remained anorexic. An appetite stimulant (mirtazapine, 1.5 mg/cat PO q24 hours) and subcutaneous fluids (100 ml of Lactated Ringers Solution) were administered, and the anorexia resolved. Topical 10% imidacloprid/1% moxidectin (Advantage Multi for Cats^®^, Bayer DVM, Shawnee Mission, KS, USA) was applied between the shoulders once monthly to treat the dirofilariasis.

Seven days after the first administration of topical 10% imidacloprid/1% moxidectin spot-on, the nodule on the ventral neck significantly decreased in size. Approximately one month after treatment, the ventral neck nodule was no longer palpable. However, a new 1.0 × 1.0 cm nodule was palpated cranial to the right clavicle and it was elected not to remove the nodule but to continue treatment with the monthly spot-on. A total of two nodules emerged over the next six-month period and all resolved. One year after resolution of all nodules, the cat remained on monthly treatment with the spot-on and no new nodules developed.

This case describes for the first time the clinical signs, diagnostics, and treatments for a domestic cat presenting with subcutaneous nodules and isolation of a *Dirofilaria* sp. most consistent with *Dirofilaria striata*. Intralesional adults and fourth stage larvae (L4) of *Dirofilaria* sp. were isolated in the present case. However, circulating microfilariae were not detected.

*Dirofilaria* spp. are filarial nematodes belonging to the Onchocercidae family (Lefoulon et al. 2015). Dirofilariasis is a mosquito-borne disease reported in several continents including, but not limited to North America, Europe, Africa, and Asia (Cringoli et al. 2007). The most studied species in dogs and cats are *D. immitis*, the causative agent of heartworm disease (Cringoli et al. 2007), and *D. repens,* the major agent of cutaneous dirofilariasis (Cringoli et al. 2007; Venco et al. 2011). Both filarial species have been reported in humans, dogs, and cats (Cringoli et al. 2007). *D. immitis* is prevalent in the USA, particularly in the Southeast and Gulf Coast states. However, it has been diagnosed in all 50 states (Cringoli et al. 2007). In contrast, *D. repens* is rarely reported in the United States, but is prevalent in Europe and Asia (Cringoli et al. 2007). In domestic cats, isolation of subcutaneous nematodes associated with skin lesions is rarely reported (Manzocchi et al. 2017). Infections of parasites morphologically identified as *D. striata* have been reported in the Southeastern United States in bobcats from Louisiana (Orihel and Ash 1964) and from Florida panthers (Forrester et al. 1985).

The life cycle of *D. striata* is not completely known. However, its genetic similarity with *D. immitis* and *D. repens* suggests that the life cycle is dependent on the infective third stage larvae (L3) and an arthropod intermediate host (Cringoli et al. 2007). Although the *Dirofilaria* sp. from this study is more genetically similar to *D. immitis* than *D. repens*, clinical manifestations more closely resemble those of *D. repens*. The biology of *D. repens* is nearly identical to *D. immitis* with the exception that maturation of L3 to adults primarily occurs within the subcutaneous connective tissues with *D. repens* compared to adult maturation in the pulmonary arteries with *D. immitis* (Cringoli et al. 2007). In dirofilariasis due to either species, monthly administration of macrocylic lactones is utilized to eliminate the microfilarial and larval stages of the parasite (Cringoli et al. 2007). In the present case, the use of monthly topical 10% imidacloprid/1% moxidectin correlated temporarily with resolution of the clinical signs and was chosen based on the labeled efficacy for *D. immitis* (Litster and Atwell 2008) and *D. repens* (Tarello 2011; ESDA 2017). However, a few nodules continued to appear during the treatment period. The relapse of nodules in this cat may be due to reinfection or survival and maturation of stages in the life cycle of *D. striata* that were not susceptible to this dose of macrocyclic lactone. In fact, while this macrocyclic lactone is able to eliminate the L3 and early L4 stages of *D. immitis*, it is not as effective against more mature stages (Nelson et al. 2018). The adult *D. immitis* may live up to 5-7 years in the canine host, with shorter lifespans typical in feline hosts (Jones et al. 2014), and treatment for adult *D. immitis* would require multiple doses of melarsomine dihydrochloride. However, this adulticidal drug is not recommended for use in cats (ESDA 2017). For this reason, it is expected that cats infected with *Dirofilaria sp*. may continue to intermittently show nodules while the various larval stages are killed, and the adults spontaneously die.

Incidentally, a *Sarcocystis* sp. sarcocyst was also identified and surgically removed from the subcutaneous connective tissue of the dorsal neck, and *Ancylostoma* spp. ova were found in the fecal flotation preparation. Both of these infections were likely secondary to the cat’s diet as a stray (i.e., feral rodents), and the cat was subclinical for these infections. In this case, serology was positive for *D. immitis* antibody but neither *D. immitis* antigen nor circulating microfilariae were detected. This is not surprising considering the cat’s lifestyle in a heartworm endemic geographic region (Miller et al. 2000; Robertson-Plouch et al. 2000).

Few deposited sequences of the *Dirofilaria* genus are currently available, and they currently include *D. immitis*, *D. repens*, and partial sequencing for *Candidatus (Dirofilaria) hongkongensis* (Pradeep et al. 2019). At the time of writing, there is no nucleotide sequence available in sequence databases to identify *D. striata*. However, parasite behavior and morphologic features in the current case match most consistently with this nematode, but without additional methodology, including intact adult worms, this cannot be confirmed.

Treatment recommendations for the current case were extrapolated from previous feline cutaneous dirofilariasis (Cornegliani et al. 2003; Cringoli et al. 2007; Venco et al. 2011) and the current guidelines for the treatment of feline heartworms (Jones et al. 2014). Macrocyclic lactones are effective for preventing *D. immitis* maturation in cats and dogs, furthermore, its use in treatment is currently being utilized in field studies for *D. repens* in Northern Italy (Cringoli et al. 2007). The use of monthly macrocyclic lactones and surgical removal has been effectively reported for feline cutaneous dirofilariasis with no reoccurrence seen after one year of treatment (Cornegliani et al. 2003). In the current case, administration of topical 10% imidacloprid/1% moxidectin and surgical removal correlated with resolution of signs of the presumed *D. striata*. However, two new nodules were seen during treatment and monthly application of topical 10% imidacloprid/1% moxidectin was continued leading to resolution of the new nodules, with no new lesions identified after 1.5 years of treatment.

In conclusion, surgical excision and monthly topical topical 10% imidacloprid/1% moxidectin correlated with resolution of nodules in this cat; future controlled studies are needed to determine treatment efficacy. Clinicians in Northern America, especially those in the Southeastern United States, should consider *D. striata* as a differential diagnosis for cutaneous nodules in cats. The sequence data obtained enables recognition of this nematode as distinct from other *Dirofilaria* spp., such as *D. immitis and D. repens.*
